# Facilitating Implementation of Research Evidence (FIRE): an international cluster randomised controlled trial to evaluate two models of facilitation informed by the Promoting Action on Research Implementation in Health Services (PARIHS) framework

**DOI:** 10.1186/s13012-018-0831-9

**Published:** 2018-11-16

**Authors:** Kate Seers, Jo Rycroft-Malone, Karen Cox, Nicola Crichton, Rhiannon Tudor Edwards, Ann Catrine Eldh, Carole A. Estabrooks, Gill Harvey, Claire Hawkes, Carys Jones, Alison Kitson, Brendan McCormack, Christel McMullan, Carole Mockford, Theo Niessen, Paul Slater, Angie Titchen, Teatske van der Zijpp, Lars Wallin

**Affiliations:** 1 Warwick Research in Nursing, Warwick Medical School, University of Warwick, Coventry, UK; 2 School of Health Care Sciences, Bangor University, Bangor, UK; 3 Fontys School of People and Health Studies, Fontys University of Applied Sciences, Eindhoven, The Netherlands; 4 School of Health and Social Care, London South Bank University, 103 Borough Road, London, SE1 0AA UK; 5 Centre for Health Economics and Medicines Evaluation (CHEME), Bangor University, Bangor, UK; 6 Faculty of Medicine and Health Science, Department of Nursing, Linkoping University, Linkoping, Sweden; 7 Department of Neurobiology, Care Sciences and Society, Division of Nursing, Karolinska Institutet, Stockholm, Sweden; 8 Faculty of Nursing, University of Alberta, Edmonton, Alberta Canada; 9 Adelaide Nursing School, University of Adelaide, Adelaide, Australia; 10 Clinical Trials Unit, Warwick Medical School, University of Warwick, Coventry, UK; 11 College of Nursing and Health Sciences, Flinders University, Adelaide, South Australia Australia; 12 Division of Nursing, Queen Margaret University, Edinburgh, UK; 13 Institute of Applied Health Research, University of Birmingham, Birmingham, UK; 14 Warwick Medical School, University of Warwick, Coventry, UK; 15 Institute of Nursing and Health Research, Ulster University, Shore Rd, Belfast, Northern Ireland; 16 School of Education, Health and Social Studies, Dalarna University, Falun, Sweden; 17 Department of Health and Care Sciences, The Sahlgrenska Academy, University of Gothenburg, Gothenburg, Sweden

**Keywords:** Facilitation, Implementation, PARIHS, Urinary incontinence, Context, Older people, RCT

## Abstract

**Background:**

Health care practice needs to be underpinned by high quality research evidence, so that the best possible care can be delivered. However, evidence from research is not always utilised in practice. This study used the Promoting Action on Research Implementation in Health Services (PARIHS) framework as its theoretical underpinning to test whether two different approaches to facilitating implementation could affect the use of research evidence in practice.

**Methods:**

A pragmatic clustered randomised controlled trial with embedded process and economic evaluation was used. The study took place in four European countries across 24 long-term nursing care sites, for people aged 60 years or more with documented urinary incontinence. In each country, sites were randomly allocated to standard dissemination, or one of two different types of facilitation. The primary outcome was the documented percentage compliance with the continence recommendations, assessed at baseline, then at 6, 12, 18, and 24 months after the intervention.

Data were analysed using STATA15, multi-level mixed-effects linear regression models were fitted to scores for compliance with the continence recommendations, adjusting for clustering.

**Results:**

Quantitative data were obtained from reviews of 2313 records. There were no significant differences in the primary outcome (documented compliance with continence recommendations) between study arms and all study arms improved over time.

**Conclusions:**

This was the first cross European randomised controlled trial with embedded process evaluation that sought to test different methods of facilitation. There were no statistically significant differences in compliance with continence recommendations between the groups. It was not possible to identify whether different types and “doses” of facilitation were influential within very diverse contextual conditions. The process evaluation (Rycroft-Malone et al., Implementation Science. doi: 10.1186/s13012-018-0811-0) revealed the models of facilitation used were limited in their ability to overcome the influence of contextual factors.

**Trial registration:**

Current Controlled Trials ISRCTN11598502. Date 4/2/10.

The research leading to these results has received funding from the European Union’s Seventh Framework Programme (FP7/2007–2013) under grant agreement no. 223646.

**Electronic supplementary material:**

The online version of this article (10.1186/s13012-018-0831-9) contains supplementary material, which is available to authorized users.

## Background

It is important that health care practice is underpinned by high quality research evidence, so that the best possible care can be delivered. However, evidence from research is not always utilised in practice [1–3]. This study used the Promoting Action on Research Implementation in Health Services (PARIHS) framework [4] as its theoretical underpinning to test whether two different approaches to facilitation could affect the use of research evidence in practice. The PARIHS framework was built upon an argument that three factors influence the uptake of research evidence in practice: the nature (strength) of the evidence, the context in which it is used, and the extent of facilitation (or help) that people have to use the evidence. The published protocol for this study [5] and an online summary report for the funder [6] contain further details.

Consistent with recent calls for an increase in theory-based implementation research [7], we used the PARIHS framework and identified two alternative types of facilitation to evaluate within the FIRE study. We chose to evaluate facilitation because whilst it is a promising approach to implementation, it has received relatively little attention and the limited results available of its effectiveness were mixed. [8–10] Facilitation has been described as a process and a role [11]. More recently it has been argued [12] “conceptual ambiguities” challenge our understanding of facilitation’s effectiveness and we do not know how to “appropriately set the degree of facilitation”. It is clear from the literature that the role and effectiveness of facilitation in implementing evidence into practice needs to be explored and tested. This study was novel in scale with a cross-country setting, and in that it sought to compare facilitation approaches that varied in terms of focus, duration and intensity.

Urinary incontinence in long-term care settings is a major issue and was thus selected as an exemplar for evaluating different approaches to implementing evidence into practice. Incontinence is a “discrediting and stigmatising” condition that affects quality of life [13]. It has a high prevalence in long-term care settings, between 40 and 70% [14], and it is a key priority within international health policy [15]. The relevance and fit of the PARIHS framework in long-term care settings for older people [16] highlighted that the factors discussed as important for change in their setting showed a good fit with those identified in the PARIHS framework, and recommended its use in these settings. We designed the FIRE trial to test two different approaches to facilitation and compare these against standard dissemination of recommendations for continence promotion [5].

### Aims

We aimed to extend knowledge of facilitation as a process for getting research evidence into practice by testing the effectiveness of and evaluating the contribution two different models of facilitation can make to implementing evidence-based urinary continence recommendations into practice.

The objectives of the study were to

1) Extend existing knowledge of facilitation as a process for translating research evidence into practice.

2) Evaluate the feasibility and effectiveness of two different models of facilitation in promoting the uptake of research-based recommendations on continence promotion, compared with standard dissemination.

3) Advance existing knowledge of guideline implementation in healthcare, with a particular focus on understanding the impact of contextual factors on the processes and outcomes of implementation.

4) Implement a pro-active dissemination strategy that complements the design of the study and facilitates the diffusion of the study findings to a wide policy and practice community throughout Europe and beyond. This objective is not considered further in this paper.

## Methods

### Design

A pragmatic cluster randomised controlled trial with embedded process and economic evaluation was undertaken. The process evaluation is reported in a linked paper (Rycroft-Malone et al. [17]).

### Participants

Staff: an internal facilitator (a member of staff from the long-term care setting) nominated in each intervention site to work with external facilitators (EFs) to implement the urinary incontinence (UI) recommendations.

Residents: aged 60 years or more with documented urinary incontinence.

### Setting

The study took place in four European countries (England, Sweden, Netherlands, Republic of Ireland), and each country planned to recruit six long-term nursing care sites (nursing homes and other residential settings with long term nursing care) (total 24 sites) for people aged 60 years or more with documented urinary incontinence. All settings had publicly funded places.

### The intervention

In arm one, the eight settings randomised to the standard dissemination control group had the urinary continence recommendations and a PowerPoint presentation on implementation (based on one utilised by Rycroft-Malone et al. [18]) sent to the head of each site. Both the intervention groups also received the same as the standard dissemination sites.

In addition, EFs prepared two different facilitator development programmes, each of which involved an initial residential programme, followed by virtual support (monthly telephone group supervision and email communication) for the internal facilitators (IFs) in implementing the UI recommendations. Arm two received a type of facilitation that we termed ‘type A’, which is a goal-focused approach to facilitation based on principles of quality improvement, management studies and organisational learning. This involved a 12 month programme for IFs nominated by each of the eight sites in this arm. This started with the IFs taking part in a 3-day residential programme run by two EFs (GH and AK), followed by 10 days over 12 months to work locally on the implementation and evaluation of recommendations, supported by 12 half-days for monthly teleconferences and self-directed study (16 days in total).

Arm three received a type of facilitation that we termed ‘type B’, which is underpinned by principles of stakeholder empowerment and overcoming external and internal obstacles to using research evidence in practice. This is achieved through the creation of workplace cultures of effectiveness in which work-based learning as inquiry is valued and supported at all levels of the organisation. This approach is informed by critical social theory and holistic facilitation. IFs nominated by each of the eight settings participated in a 24-month development programme. This started with a 5-day residential programme run by two EFs (BMcC and AT) followed by 20 days to work on the local implementation and evaluation of the recommendations, supported by 24 half-day learning groups via teleconferencing, and 12 half-days for self-directed study (38 days in total). The EFs each have over 20 years’ experience of facilitation. Additional file 1: File S1 contains more details on the underpinning theories and activities in each intervention.

A model of co-facilitation was used in both facilitation arms where a second staff member in the organisation, a “buddy”, worked with the IF, using this as a development opportunity, including taking the lead if the initial facilitator was unable to continue.

### Outcomes measures

The primary outcome was the documented percentage compliance with continence recommendations produced by the fourth International Consultation on Incontinence [19]. Percentage compliance is calculated for each resident, so it is measured at the resident level.

These recommendations included (1) the resident should be actively screened for incontinence (five components), (2) a detailed assessment should be carried out (15 components), (3) an individualised treatment plan should be in place (13 components) and (4) a specialist referral should be made if needed (one component). These outcomes were assessed at baseline, then at 6, 12, 18 and 24 months. Additional file 2: File S2 lists all components of the continence recommendations.

#### Secondary outcomes

Included the documented incidence of level of cognitive impairment (as this influences the type of continence care the guidance recommends), depression, incontinence-related dermatitis, urinary tract infections (UTIs), health-related quality of life (EQ-5D [20] and IQoL [21]) and the proportion of residents in the setting with incontinence and use of pelvic floor exercises. Organisational context was assessed using the Alberta Context Tool (ACT) [22, 23]. The ACT data was collected from Nurses, Licenced Practical Nurses (LPN) and Health Care Assistants (HCA) at baseline in 23 of the 24 sites.

#### Sample size and power calculations

There was no information on existing compliance with the continence recommendations. We took a 50% compliance as an initial assumption. It was assumed that each setting would have 50 residents available for assessing compliance. For 90% power to detect compliance of 15% better in the intervention compared to control arm and allowing for an intra-cluster correlation of 0.01 (typically found in Primary Care Studies [24]) and statistical tests carried out at the 5% level, for a cluster size of 50, seven clusters (long term care settings) were required per intervention arm. Thus 7 × 3 arms = 21 clusters were needed. Allowing for potential attrition, this was increased to 8 clusters per arm, so 24 clusters in total. This equates to 6 long-term nursing care settings in each of the four countries with 50 or more residents per setting. Consent was sought at cluster and at individual level, the former before randomisation and the latter after randomisation.

### Randomisation sequence generation, allocation concealment, implementation and blinding

In each country, sites were randomly allocated to one of three arms (standard dissemination, and two different intensities and kinds of a facilitation intervention), using a random sequence generated by the statistician. A centralised randomisation point was set up by the study statistician to ensure allocation concealment. Long-term care settings were enrolled by country leads for the study. The statistician was blinded to the intervention group. It was not possible to blind site staff to intervention. Research fellows who collected data from records and where necessary obtained consent from residents were blinded to the intervention group, but as discussed in the protocol [5], previous experience suggested this blinding may be inadvertently broken by the sites.

#### Quantitative analysis—statistical methods

Data were analysed using STATA15. The primary outcome measures, percentage compliance, were analysed by fitting multi-level mixed-effects linear regression models with standard errors adjusted for the clustering at the level of the nursing care setting (site level) [25]. Data was collected every 6 months, but because the resident population was constantly changing it is necessary to consider the data as repeated cross-sectional assessments of residents in the care settings rather than longitudinal assessment of individuals within the care settings. The regression models include three independent variables: study arm (three levels), country (four levels), time period (five levels baseline, 6 months, 12 months, 18 months, 24 months), interaction terms would only be fitted if study arm main effects were significant. Intra-cluster correlation coefficients (ICC) for the baseline measurements of the compliance scores were calculated through ANOVA with adjustment for clustering and unequal cluster size. Post-estimation ICCs are calculated after fitting the regression models. Descriptive statistics, ANOVA and chi-square tests were used, where appropriate, to examine differences between groups with regard to secondary outcomes. Data were examined by an independent data monitoring committee.

#### Qualitative analysis

The process evaluation data were analysed from a realist perspective [26] and are reported in the linked paper (Rycroft-Malone et al. [17]).

### Findings

In each country, we planned to recruit six sites (two sites per arm). This happened for Sweden and the Netherlands. However, one site in England withdrew before the study started. When no additional site was forthcoming in England within the timeframe, an additional control site was recruited in Republic of Ireland and ethical clearance obtained. This final site had data collected up to month 18 only as there was not time to collect data at 24 months. There were thus five sites in England (with one site in the control arm) and seven in the Republic of Ireland (three sites in the control arm). Each cluster (site) received the allocated intervention and were analysed for primary and secondary outcomes.

Quantitative data were available from 2313 resident records across all time points (*n* = 430 at baseline, *n* = 462 at 6 months, *n* = 497 at 12 months, *n* = 479 at 18 months and *n* = 445 at 24 months after the intervention). The sample is described and then the primary outcome, compliance with the four continence recommendations is presented. The study took place between 2010 and 2013.

#### Description of resident sample

Most residents were included at one time point only. In all four countries, at baseline the mean age of residents varied from 82 to 87 years. This was almost unchanged at 24 months later (range 82–86 years). In all four countries, there were more female than male residents at baseline (the percentage female in each site ranged from 60 to 71%), and this was similar at 24 months (range 54–80% females). At baseline, the mean age of residents allocated to the three intervention groups was very similar (control 85.34 years (s.d. 7.39); type A 86.35 years (s.d. 7.19); type B 83.20 years (s.d. 8.48)). The gender mix was also similar for the three intervention groups (control 68.8% female; type A 62.2% female; type B 73.8%).

To understand the health status of the residents, data from the EQ-5D visual analogue scale (VAS) measure of health state that we administered at 24 months provides summary information for each intervention group (Table 1)*.* Data at 24 months are chosen because EQ-5D-VAS was available for a higher proportion of residents than any other time point. Higher scores on a scale of 0–100 represent better health states. Table 1 shows there was no significant difference in the mean EQ-5D scale for the intervention groups; so on average resident health status was similar in all the intervention groups. Not all residents were able to complete or have a proxy complete an EQ-5D so numbers completed are lower than total number of residents.

**Table 1:** Summary statistics for EQ-5D-VAS scale for each intervention group

Intervention	Number of residents with completed scale	Mean (SE robust)^a^	95% CI for mean	Range
Standard dissemination (control)	109	54.2 (4.737)	44.35, 64.00	0, 100
Type A	113	59.2 (4.325)	50.19, 68.13	0, 100
Type B	124	55.6 (2.918)	49.57, 61.67	0, 90

^a^SE robust allows for the clustering, and ANOVA allowing for clustering to compare the three means, gave *p* = 0.34

### Primary outcomes—compliance with the four continence recommendations

(Full details of all the components of each of the four continence recommendations are available in the Additional file 1: File S1). The ICC for percentage compliance with recommendations has been calculated from the baseline data, making allowance for both the clustering and the unequal numbers from the 24 long-term care settings. The ICC for percentage compliance with recommendation 1 is 0.545 (95% CI 0.361, 0.730); for percentage compliance with recommendation 2, the ICC is 0.404 (95% CI 0.220, 0.587), and for percentage compliance with recommendation 3, ICC was 0.455 (95% CI 0.270, 0.641). These ICCs are much higher than expected and those usually found in Primary Health Care studies of 0.01 [24], they are more similar to those found in some educational cluster trials [27].

The results reported in Tables 2, 4 and 6 are from fitting multi-level mixed-effect linear regressions models to the compliance scores for each of the recommendations 1, 2 and 3 respectively. These models account for the cluster design by treating site as a random effect and adjusting the standard error for the 24 site clusters. The model includes three independent variables: study arm (three levels—control, type A and type B), country (four levels—Netherlands, Sweden, Republic of Ireland and England), time period (five levels—baseline, 6 months, 12 months, 18 months and 24 months). The first level for each variable (control arm for intervention, Netherlands for country and baseline for time) are taken as the base level and other levels are compared to this. In this model, we are considering the effect of intervention allowing for country and time. The assumptions of linear regression were examined, and there was no evidence that the data failed to meet these assumptions. As a sensitivity analysis, the linear regression models were also fitted omitting the country covariate, and this did not change any of the findings with regard to the significance of the intervention effect.

**Table 2:** Multi-level mixed-effect linear regression model—percentage compliance with recommendation 1 (the resident should be actively screened for urinary incontinence), with adjustment of standard errors to allow for clustering

	Coefficient	Std. Err.	*z*	*p* value	95% confidence interval
Type A	2.9293	3.1298	0.94	0.349	− 3.2049, 9.0635
Type B	− 4.1688	3.6966	− 1.13	0.259	− 11.4141, 3.0765
Sweden	− 31.0840	4.0940	− 7.59	0.000	− 39.1082, − 23.0599
Ireland	13.8449	4.5226	3.06	0.002	4.9808, 22.7091
England	10.3152	5.0742	2.03	0.042	0.3699, 20.2604
+ 6 months	0.1104	2.8436	0.04	0.969	− 5.4630, 5.6837
+ 12 months	12.9885	4.4264	2.93	0.003	4.3130, 21.6641
+ 18 months	4.9052	3.3204	1.48	0.140	− 1.6025, 11.4130
+ 24 months	9.3776	4.3632	2.15	0.032	0.8259, 17.9292
Constant	33.7259	4.2278	7.98	0.000	25.4396, 42.0122

*N* = 2313; model fit: Wald *χ*^*2*^ (9)=1970.23, *p* < 0.001; post-estimation ICC 0.0910 (se 0.0219)

### Compliance with recommendation 1: The resident should be actively screened for urinary incontinence

Compliance with recommendation 1 can range from 0 to 5 depending on which of five potential components of this recommendation are documented. For each component documented, one point is scored, percentage compliance is a score out of 5 as a percentage. Table 2 reports the model for compliance with recommendation 1 and shows outcome scores in the intervention arms did not reach statistical significance. Country is significant with Sweden having poorer compliance (a negative coefficient) compared to the Netherlands. Ireland and England had significantly better compliance than the Netherlands (positive coefficients). The 12- and 24-month data collection parameters were significant, but the other points were not significantly different to baseline. The post-estimation ICC following the fitting of this model for compliance with recommendation 1 is 0.091. Table 3 shows the mean percentage for each intervention group at each time point, showing the small increase in percentage compliance score for type A and type B intervention up to 12 months, though as the regression model indicates there is no significant difference in the study arms over the duration of the study.

**Table 3:** Mean percentage compliance with recommendation 1 by intervention group for each time point

Intervention group	Mean score				
	Baseline	6 months	12 months	18 months	24 months
Control	28.4	22.3	29.2	27.0	23.2
Type A	19.2	21.5	38.8	30.6	35.5
Type B	14.1	17.0	44.4	23.4	28.7

*N* = 2313 residents are included in this analysis

### Compliance with recommendation 2: A detailed assessment should be carried out

There are 15 items in the detailed assessment, so scores can range from 0 to 15 for recommendation 2. Percentage compliance is a score out of 15 as a percentage.

Table 4 reports the fitted model for compliance with recommendation 2. The intervention is not effective; neither the type A facilitation or type B facilitation interventions had significant coefficients. Ireland was significantly different having higher compliance with recommendation 2, but the coefficients for the other countries were not significant, so England and Sweden are not significantly different to the Netherlands after allowing for time point and intervention group. The 24-month data collection parameter is significant, with increased compliance by 24 months, but the other points are not significantly different to baseline. The post-estimation ICC following the fitting of this model for compliance with recommendation 2 is 0.351. Table 5 shows mean percentage compliance score for recommendation 2 by intervention group. Mean percentage compliance was low at baseline, in all groups, but improved by 24 months in the type A and type B intervention groups.

**Table 4:** Multi-level mixed-effect linear regression model—percentage compliance with recommendation 2 (a detailed assessment should be carried out), with adjustment of standard errors to allow for clustering

	Coefficient	Std. Err.	*z*	*p* value	95% confidence interval
Type A	5.6514	4.0014	1.41	0.158	− 2.1912, 13.4941
Type B	3.7903	4.4807	0.85	0.398	− 4.9917, 12.5724
Sweden	− 1.9108	2.7374	− 0.70	0.485	− 7.2760, 3.4545
Ireland	14.9312	3.6627	4.08	0.000	7.7524, 22.1099
England	11.7997	7.0278	1.68	0.093	− 1.9745, 25.5738
+ 6 months	− 0.2220	1.2763	− 0.17	0.862	− 2.7235, 2.2794
+ 12 months	3.3623	2.1118	1.59	0.111	− 0.7767, 7.5014
+ 18 months	− 0.0031	1.6463	− 0.00	0.998	− 3.2298, 3.2235
+ 24 months	4.4827	2.1665	2.07	0.039	0.2364, 8.7290
Constant	30.1617	3.3204	9.08	0.000	23.6538, 36.6696

*N* = 2313; model fit: Wald *χ*^*2*^ (9) = 64.76, *p* < 0.001; post-estimation ICC 0.3517 (se 0.0758)

**Table 5:** Mean percentage compliance with recommendation 2 by intervention group for each time point

Intervention group	Mean score				
	Baseline	6 months	12 months	18 months	24 months
Control	37.5	34.6	36.5	34.1	34.4
Type A	34.6	35.1	45.1	39.7	44.6
Type B	35.3	34.8	43.2	38.2	45.9

*N* = 2313 residents are included in this analysis

### Compliance with recommendation 3: An individualised treatment plan should be in place

A score from 0 to 13 is possible for compliance with recommendation 3. Percentage compliance is a score out of 13 as a percentage.

Table 6 reports the fitted model for compliance with recommendation 3. The intervention was not effective, neither the type A facilitation or type B facilitation interventions had significant coefficients. All country parameters were significant with Sweden, Ireland and England all having significantly higher compliance with recommendation 3 than the Netherlands. All time points were significant, and the parameter value increased for each successive time period, thus suggesting improvement over time in compliance with recommendation 3. This suggests learning over time in all countries, but no significant difference in the effectiveness of the three study interventions. The post-estimation ICC following the fitting of this model for compliance with recommendation 3 is 0.126. Table 7 shows mean percentage compliance for recommendation 3 by intervention group. It can be seen that all three groups appear to improve over time, with little difference between the interventions as indicated by the regression model.

**Table 6:** Multi-level mixed-effect linear regression model—percentage compliance with recommendation 3 (an individualised treatment plan should be in place), with adjustment of standard errors to allow for clustering

	Coefficient	Std. Err.	*z*	*p* value	95% confidence interval
Type A	0.3391	4.0168	0.08	0.933	− 7.5336, 8.2118
Type B	1.0372	3.0579	0.34	0.734	− 4.9562, 7.0305
Sweden	23.7959	1.9736	12.06	0.000	19.9278, 27.6640
Ireland	24.5448	3.9162	6.27	0.000	16.8692, 32.2204
England	15.3118	3.8489	3.98	0.000	7.7681, 22.8555
+ 6 months	9.8431	4.1862	2.35	0.019	1.6382, 18.0479
+ 12 months	14.2761	3.7488	3.81	0.000	6.9285, 21.6237
+ 18 months	15.9399	3.7804	4.22	0.000	8.5305, 23.3494
+ 24 months	19.9791	3.3984	5.88	0.000	13.3183, 26.6399
Constant	6.5831	3.0927	2.13	0.033	0.5216, 12.6446

*N* = 2313; model fit: Wald *χ*^*2*^ (9)=387.72, *p* < 0.001; post-estimation ICC 0.1265 (se 0.0502)

**Table 7:** Mean percentage compliance with recommendation 3 by intervention group for each time point

Intervention group	Mean score				
	Baseline	6 months	12 months	18 months	24 months
Control	20.9	30.8	40.9	45.0	48.9
Type A	23.8	32.2	41.9	42.7	45.2
Type B	26.7	38.6	40.7	41.1	45.9

*N* = 2313 residents are included in this analysis

### Recommendation 4: Specialist referral should be made if necessary

There were very few specialist referrals made and in the data collection it was not always clear whether a lack of documentation meant no referral was made or whether a referral was not necessary. It is therefore difficult to fully assess compliance with this guideline. However, the level of referral was so low that it is very unlikely that study arm has a significant impact on compliance with this recommendation. In only 4% of residents was a referral recommended. Although these referrals were recorded as specialist referrals, 17 were to a general practitioner (family doctor) and 6 to an unknown specialist. There were only 11 referrals to a continence specialist nurse and 6 referrals to urology.

In summary, for the primary outcome (documented compliance score or percentage compliance with continence recommendations), there was no significant difference between study arms; all study arms improved over time in all countries.

### Secondary (clinical) outcomes

These data are being considered as two cross-sectional reviews of the resident populations in the long-term care settings as there are very few individual residents included at both baseline and 24 months data collection. At 24 months, there was no significant difference between the three intervention groups with regard to the proportion of residents who had no documented record of the assessment of cognition (*p* = 0.076 from chi-square test). At 24 months, there was a significant difference between the three intervention groups with regard to the proportion of residents who had no documented record of the level of cognitive impairment (*p* < 0.001 from chi-squared test), the proportion being higher in the control group than in the type A and type B groups. At 24 months, there was a significant difference between the three intervention groups with regard to the proportion of residents who had no documented record of the assessment of depression (*p* = 0.017 from chi-squared test), the proportion being higher in the control group than in the type A and type B groups. At 24 months, there was no significant difference between the three intervention groups with regard to the proportion of residents who had no documented record of the assessment of incontinence-related dermatitis (*p* = 0.479 from chi-square test).

Between baseline and 24 months, there was a statistically significant decrease in the proportion of residents who had no documented record of an assessment of cognition in type B facilitation (*p* < 0.001) but no significant change for type A; there was a significant decrease in the proportion of residents who have no documented record of the level of cognitive impairment in intervention type A (*p* < 0.001) and type B (*p* < 0.001); there was a significant reduction in the percentage of residents who had no documentation of assessment of depression in the type A (*p* < 0.001) and type B (*p* < 0.001) groups. There was a significant decrease in the percentage of residents who had no documentation of incontinence-associated dermatitis between baseline and 24 months in the type A (*p* < 0.001) and type B (*p* < 001) groups. There was no significant improvement in the control group for any of the secondary outcomes.

Whether the impact of urinary incontinence on quality of life been assessed was not documented for the majority of residents. It was not assessed more than seven times in any group, so this was not explored further. Very few UTIs were documented. In the month prior to the baseline data collection, only 15 UTIs were recorded in all countries, decreasing to only seven at the 24 month data collection point. No further analysis was done.

It was not possible to reliably calculate the proportion of residents in each long-term care setting with incontinence, thus no further analysis was done. At baseline, pelvic floor exercises were not used with any residents, and at 24 month follow up pelvic, floor exercises were only used with three residents. With such low numbers, no further exploration of this is sensible.

In summary, for secondary outcomes, both the facilitation intervention groups (type A and type B) showed significantly better documentation of three outcomes: the level of cognitive impairment, depression and incontinence-associated dermatitis between baseline and 24 months, and this improvement did not occur in the standard dissemination (control) group. Clinically, this change was not large, and a substantial proportion of residents still had no documented assessment of level of cognitive impairment (68% in type A and 65% in type B) depression (61% in type A and 65% in type B) and incontinence-associated dermatitis (66% in type A and 73% in type B).

There was a large amount of missing data on the Urinary Incontinence Quality of Life (I-QoL) outcome measure [21] as residents found it too much to complete, so this is not reported further. It had been planned to report length of stay data, but it was not possible to collect this data consistently across all sites, so it is not reported further.

### Health economics

Health economic analysis was undertaken, but since there was no significant difference in the primary outcome between the intervention groups, these data are not presented here in detail because the cost analysis showed that, as expected, standard dissemination would be the least costly intervention to implement. (see Additional file 1: File S3 for intervention cost tables).

### Alberta Context Tool

For all concepts, higher scores represent a better work context. All responses for a site (Nurse, LPN, HCA) were considered together to provide an overall picture of the site. The questionnaire completed by Nurses, LPN’s and HCAs were identical except with regard to informal interactions in which the HCA group had one less question (9) than the other groups of staff who had 10 questions in this section.

Table 8 shows for each concept the mean score given by all staff rating a site within the intervention arm. Formal interactions are notably lower than other scores. The largest differences are for structural and electronic resources and for organisational slack-space. On the basis of the similarity of these mean scores, we conclude the study groups were similar with regard to ACT concepts.

**Table 8:** Mean scores on ACT concepts by intervention group at baseline (*N* = 725 staff are included in this analysis)

ACT concept^a^	Number of items	Range for score	Control sites Mean (SD)	Type A sites Mean (SD)	Type B sites Mean (SD)
Leadership^b^	6	1–5	3.6 (0.81)	3.7 (0.82)	3.7 (0.76)
Culture^b^	6	1–5	3.9 (0.65)	3.9 (0.57)	3.9 (0.61)
Feedback^b^	6	1–5	3.5 (0.79)	3.4 (0.82)	3.4 (0.85)
Formal interactions^c^	4	0–4	1.3 (1.14)	1.1 (1.08)	1.2 (1.13)
Informal interactions^c^	9 or 10	0–10	3.5 (2.11)	3.2 (2.08)	3.3 (2.04)
Connections (social capital)^b^	6	1–5	4.0 (0.67)	3.8 (0.59)	3.9 (0.59)
Structural and electronic resources^c^	11	0–11	3.1 (2.34)	3.4 (2.14)	2.8 (1.89)
Organisational slack-staffing^b^	3	1–5	2.7 (1.13)	2.8 (1.09)	2.6 (1.00)
Organisational slack-space^b^	3	1–5	3.6 (1.01)	3.1 (1.14)	3.3 (1.10)
Organisational slack-time^b^	4	1–5	2.8 (0.69)	2.8 (0.70)	2.8 (0.74)

^a^Definitions of ACT concepts and scaling are provided [21, 22], and relevant papers are listed at https://trecresearch.ca/alberta_context_tool

^b^Scaled

^c^Count based

## Discussion

The 12 months type A and the 24 months type B facilitation interventions did not have different levels of impact on documented compliance with recommendations. It was thus not possible to identify the type and “dose” of facilitation that worked best within the highly varied contextual conditions identified in this study. In addition, the process evaluation revealed important issues about the models of facilitation used and the characteristics of the facilitators [17, 28].

So why was it that the facilitation intervention did not make a statistically significant difference to the documented implementation of continence recommendations? Was an element of the PARIHS framework, facilitation, purported to be necessary for getting research evidence used in practice, actually not so important? Other research has found some type of help with getting research implemented does make a difference [29, 30]. Baskerville et al.’s [31] systematic review of practice facilitation in primary care suggests facilitation improves uptake of clinical practice guidelines by nearly three times. A facilitation intervention was found to reduce neonatal mortality by 50% [32]. Although the facilitation not working in this study is a possible explanation and the high ICCs meant the study was underpowered, the process evaluation qualitative research evidence [17] suggested this was not the most likely explanation. It may be facilitation works differently along the continuum of context. It could be that using only documented evidence of compliance with the recommendations under-estimated what might have happened in practice but was not documented. A lack of intervention fidelity is another possible explanation [17].

Although the intervention groups improved, it was not possible to say the improvement was due to the intervention as the control group also improved. We do not know why this was, but it could be that for control sites, being in the study, including six monthly follow-ups for 2 years, was enough of an incentive to improve. However, the qualitative data suggests for most control sites they did not use the written recommendations or the implementation guide. One site mentioned to the researcher that they checked their documentation and practice knowing the researcher would be visiting, and thus even collecting follow-up data in the control group can be seen as having an effect.

Etheridge et al. [33] concluded that four active ingredients were required to effect change in long-term care settings: urgency, solidarity, intensity and accumulation. The continence programme they reviewed failed, and one of the reasons they identified may also apply in our study: there was no buy-in from participants. Although all sites agreed to take part in the study, the topic area and the intervention were already decided. In addition, participants changed during the study, so, for example, as managers changed, new managers did not necessarily see this study as a priority, thus reducing even further the extent of organisational buy-in and support [17].

The proposition that underpins the PARIHS framework is that successful implementation is a function of the nature of the evidence being implemented, the context into which it is being implemented and appropriate facilitation to help people implement the evidence. There was no weighting given to these three aspects of evidence, context and facilitation. This research suggested that facilitation with one or two people in a team may not easily overcome contextual factors. The level of experience and expertise of the IF, and relationship of the IF to managers in the setting, may be more important [34] as may unravelling how facilitation and context interact.

It was not possible to identify a “good enough” model of facilitation that affected the primary outcome (documented compliance with continence recommendations) and could address the different contexts. Facilitation did however result in some identifiable practice changes (e.g. new assessment processes, new forms and awareness of the impact of incontinence on residents).

It may be that in practice, tailoring the type of facilitation to both the setting and the internal facilitator is important. Just how one could map the contextual characteristics to a type of facilitation and to type of internal facilitator would need further evaluation. Van der Zijpp et al. [34], part of this study, argued the interactions between managerial leaders and IFs were important, summarised by three themes: realising commitment, negotiating conditions and encouraging to keep the momentum going. The reciprocal relationships between managers and IFs influenced the process of implementation, and future interventions should target managers in a focused way. In studies that evaluate implementation of complex interventions such as facilitation, it may be appropriate to adopt a theoretical perspective on fidelity, focusing on the intended mechanisms of the intervention. For example, in this study, the theory of type A facilitation required IFs to develop skills and confidence in audit and feedback. Achieving this mechanism, even if it meant IFs needed varying levels of external facilitation, would demonstrate theoretical fidelity. This type of approach has been proposed in public health [35] and is discussed in the linked papers (Rycroft-Malone et al. [17], Harvey et al. [28]).

ACT considers organisational concepts as a unit-based score. In this study, these were considered as site level variables. Mean baseline and follow-up mean scores were compared with either an ANOVA where multiple time points were available or with a t-test when only one follow-up time point was available. There were very few changes that were significant. We are thus not confident to make any claims about the effects of the intervention on organisational culture as assessed with ACT. Possible explanations for this include the organisations were stable and at site level the concepts were unaffected by the interventions.

### Limitations

In reality, the planned interventions did not always work as originally envisaged, as revealed by the process evaluation [17] and our analysis of the facilitation intervention [28]. This was for several reasons, relating to initial selection and preparation of the IFs; engagement in the facilitation intervention; ability to progress according to plan. The linked papers illustrate the issues that compromised the fidelity of the intervention [17, 28]. It was also challenging to recruit resident participants in some homes, so we had fewer than planned. In addition, although each of the long-term settings had agreed to take part, for individual staff within the home it was not necessarily a priority. The unexpectedly high ICC meant the study was underpowered. Although we felt the ICC we used in the sample size calculation was reasonable, in planning future cluster RCTs with a more educational focus, it is important to be aware that not all ICCs will be as low as those reported for recent primary care trials [24]. In the design of the study, it was assumed that there would not be large country differences regarding compliance with the recommendations; hence, it would be viable to have a small number of sites from each country in each study arm. In practice, it appears the countries are behaving differently, but the study was not powered to investigate within country effect of the different interventions on the primary outcome.

## Conclusions

Pressman and Wildavsky [36] a long time ago reported that “the study of implementation requires understanding that apparently simple sequences of events depend on complex chains of reciprocal interaction” (pxvii) and referred to the complexities of implementation as “the lumpy stuff of life” [37] (p165). This study supports those assertions.

This was the first cross European randomised controlled trial with embedded process and economic evaluations that sought to test different methods of facilitation. There was no significant difference in the primary outcome between any of the three study arms. It found both models of facilitation were broadly viable but were not significantly better than a control in improving documented compliance with recommendations to promote continence. Contextual issues were not always overcome by the approaches to facilitation adopted in this study as our linked papers demonstrate (Rycroft-Malone et al., Harvey et al. [17, 28]).

## Additional file

Additional file 1: File S1 Underpinning theories and activities of type A and type B facilitation. File S2 List of all components of the continence recommendations. File S3 FIRE programme costs. File S4 Study Flow Diagram, Consort Checklist and Tidier checklist. (DOCX 71 kb)

## Abbreviations

EF: External facilitator
FIRE: Facilitating Implementation of Research Evidence
IF: Internal facilitator
PARIHS: Promoting Action on Research Implementation in Health Services
UI: Urinary incontinence
UTI: Urinary tract infection
