# Supplementary material for: Facilitating Implementation of Research Evidence (FIRE): an international cluster randomised controlled trial to evaluate two models of facilitation informed by the Promoting Action on Research Implementation in Health Services (PARIHS) framework
Source: Implement Sci. 2018 Nov 16;13:137. doi: 10.1186/s13012-018-0831-9 (PMC6238407; doi:10.1186/s13012-018-0831-9)
Supplement: Supplementary file 1 — File S1 Underpinning theories and activities of type A and type B facilitation. File S2 List of all components of the continence recommendations. File S3 FIRE programme costs. File S4 Study Flow Diagram, Consort Checklist and Tidier checklist. (DOCX 71 kb) [file 13012_2018_831_MOESM1_ESM.docx]

Additional file 1

**File S1** Underpinning theories and activities of Type A and Type B facilitation.

(This replicates Table 6 in Rycroft-Malone et al’s linked paper)

|  | **Underpinning theories** | **Activities evident of facilitation type** |
| --- | --- | --- |
| **Type A** | Quality improvement, organisational learning, and humanistic psychology – how individuals learn and apply that knowledge to improvement activities  Within the PARIHS framework type A represents an approach to facilitation towards the left of the facilitation continuum (Harvey et al 2002). | - Set up project group. - Developed action plans. - Developed posters & fliers about the project. - Audit – identify what needed to improve in continence practice. - Presentation of data in poster. - Development of information leaflets. - Development of new continence assessment forms. - Development of continence care plan. - Supported staff to complete the assessment forms. |
| **Type B** | Critical social sciences, focussed on enlightenment, empowerment and emancipation – that enable individuals to develop new understandings about what needs to be changed and how to change it, including 1) understanding, 2) choosing and development appropriate strategies, 3) doing and 4) evaluation.  Within the PARIHS framework type B represents an approach to facilitation towards the right of the facilitation continuum (Harvey et al 2002). | - Formed a project group of stakeholders. - Values clarification exercise. - Self-administered leadership questionnaires. - 360˚ feedback from colleagues. - Asked staff to complete Context Assessment Index. - Provision of person-centred care presentations to staff. - Interviewing residents with urinary continence. - Using stakeholder group to identify priorities, agree actions, evaluate progress. - Reviewed practice, revision of policies, including assessment forms. |

File S2 - List of all components of the continence recommendations

**Assessment of Recommendation Use**

**Adapted from Royal College of Physicians (United Kingdom) National Audit of Continence Care for Older** **People. (2005), (2010); used with permission.**

**The four continence recommendations**

**1) The patient/resident should be actively screened for urinary incontinence. (Five components)**

i) Does the patient have documented evidence of a continence history? Does this include an assessment of:

ii) Daytime incontinence

iii) Nocturnal incontinence

iv) Urinary frequency [> 8 voids /24 hours]

v) Nocturnal frequency [> 2 voids/night]

**2) A detailed assessment should be carried out. (15 components)**

i) Constipation

ii) Mobility problems

iii) Problems transferring

iv) Problems with dexterity

v) Problems with ability to toilet

vi) Screened for depression

vii) Screened for cognitive impairment

viii) Any medications that could cause or worsen UI

ix) Any medical conditions that could cause or worsen UI (vaginal, prostate, faecal elimination problems, tumours )

x) Has a urinalysis been documented

xi) Has a rectal examination been documented

xii) Have wet checks been documented

xiii) Has a bladder chart been used

xiv)Has fluid intake been assessed

xv) Is there documented evidence of the type of urinary incontinence

**3) An individualised treatment plan should be in place. (13 components)**

i) Is there documented evidence of a specific management plan for continence problem

ii) Does the plan include individualised goals of care

iii) Is progress against individual goals documented

iv) Is there a documented discussion with the resident about cause and management of UTI

v) Is there a documented discussion with the resident about management preferences

vi) Is there documented evidence of a discussion with the next of kin about cause and management of UI

vii) is there documented evidence of a discussion with the next of kin about management of UI preferences (can include preference for no treatment)

viii) Is there documented evidence that the resident and/or next of kin has been given a copy of the UI management plan.

ix) Is there documented evidence of a tailored prompted voiding if the resident is frailer and more impaired.

x) Is there documented evidence of bladder retraining if resident more fit and alert

xi) If wearing incontinence pads, have they had a specialist assessment

xii) Has the resident’s view of the degree of bother cause to them by UI been documented

xiii) Is the next-of kin’s view on the degree of bother to the resident been documented

**4) Specialist referral should be made if necessary. (One component)**

**File S3 -** FIRE programme costs

| Task | Arm 2 (Type A facilitation) annual cost | Arm 3 (Type B facilitation) annual cost |
| --- | --- | --- |
| EF programme development time | €6,539 | €17,175 |
| Audit system | €1,841 | N/A |
| Travel and accommodation | N/A | €1,012 |
| Materials | N/A | €223 |
| *Total development cost* | *€8,380* | *€18,410* |
| EF course preparation and delivery time | €8,322 | €16,009 |
| EF follow-up time | €3,119 | €8,363 |
| EF travel and accommodation | €2,363 | €996 |
| Materials | €427 | €1,132 |
| Admin | €2,817 | €2,273 |
| Venue hire | €4,199 | €3,896 |
| Translator expenses | €1,406 | €1,037 |
| IF travel and accommodation | €1,237 | €425 |
| Critical companion time supporting IFs | N/A | €2,355 |
| Critical companion travel | N/A | €130 |
| IF time within care homes | €8,253 | €8,805 |
| *Total running cost* | *€32,143* | *€45,421* |
| Total cost | €40,522 | €63,831 |
| Cost per home | €5,065 | €7,979 |

Note: IF: internal facilitator, EF: external facilitator; N/A is given if a cost was not applicable for the intervention arm

**File S4 – Study Flow Diagram, Consort Checklist and Tidier checklist**

Study Flow diagram

**Allocation**

**Control**

8 sites

(147 residents)

**Type A**

8 sites

(151 residents)

**Type B**

8 sites

(132 residents)

**Randomised (n=24 long term care sites)**

**Follow-up**

**Control**

No sites lost to follow-up at 6,12, 18 & 24 months *

**Type A**

No sites lost to follow-up at 6,12, 18 & 24 months

**Type B**

No sites lost to follow-up at 6,12, 18 & 24 months

**Analysis**

**Control**

6 months: 154 residents

12 months: 159 residents

18 months: 164 residents

24 months: 157 residents

**Type A**

6 months: 162 residents

12 months: 178 residents

18 months: 161 residents

24 months: 143 residents

**Type B**

6 months: 146 residents

12 months: 160 residents

18 months: 154 residents

24 months: 145 residents

********

* 1 control site recruited late so data only available to 18 months in that site.

**CONSORT 2010 checklist of information to include when reporting a cluster randomised trial**

| Section/Topic | Item No | Standard Checklist item | Extension for cluster designs | Page No * |
| --- | --- | --- | --- | --- |
| Title and abstract | | | |  |
|  | 1a | Identification as a randomised trial in the title | Identification as a cluster randomised trial in the title | p4, lines 49-51 |
|  | 1b | Structured summary of trial design, methods, results, and conclusions (for specific guidance see CONSORT for abstracts)^[[1]](#endnote-1),^^[[2]](#endnote-2)^ | See table 2 | p4-5, lines 52-78 |
| Introduction | | | |  |
| Background and objectives | 2a | Scientific background and explanation of rationale | Rationale for using a cluster design | p6-7, lines 88-116 |
|  | 2b | Specific objectives or hypotheses | Whether objectives pertain to the cluster level, the individual participant level or both | p7, lines 121-131 |
| Methods | | | |  |
| Trial design | 3a | Description of trial design (such as parallel, factorial) including allocation ratio | Definition of cluster and description of how the design features apply to the clusters | p8-9, lines 134-147 |
|  | 3b | Important changes to methods after trial commencement (such as eligibility criteria), with reasons |  | N/A |
| Participants | 4a | Eligibility criteria for participants | Eligibility criteria for clusters | P8, lines 168-171137-141 |
|  | 4b | Settings and locations where the data were collected |  | P8, lines 142-146 |
| Interventions | 5 | The interventions for each group with sufficient details to allow replication, including how and when they were actually administered | Whether interventions pertain to the cluster level, the individual participant level or both | P8, lines 147-175 |
| Outcomes | 6a | Completely defined pre-specified primary and secondary outcome measures, including how and when they were assessed | Whether outcome measures pertain to the cluster level, the individual participant level or both | P9-10, lines 1176-190 |
|  | 6b | Any changes to trial outcomes after the trial commenced, with reasons |  | N/A |
| Sample size | 7a | How sample size was determined | Method of calculation, number of clusters(s) (and whether equal or unequal cluster sizes are assumed), cluster size, a coefficient of intracluster correlation (ICC or *k*), and an indication of its uncertainty | P10, lines 191-200 |
|  | 7b | When applicable, explanation of any interim analyses and stopping guidelines |  | N/A |
| Randomisation: | | | |  |
| Sequence generation | 8a | Method used to generate the random allocation sequence |  | P10-11, lines 1201-208 |
|  | 8b | Type of randomisation; details of any restriction (such as blocking and block size) | Details of stratification or matching if used | P10-11, lines 201-208 |
| Allocation concealment mechanism | 9 | Mechanism used to implement the random allocation sequence (such as sequentially numbered containers), describing any steps taken to conceal the sequence until interventions were assigned | Specification that allocation was based on clusters rather than individuals and whether allocation concealment (if any) was at the cluster level, the individual participant level or both | P10-11, lines 201-208 |
| Implementation | 10 | Who generated the random allocation sequence, who enrolled participants, and who assigned participants to interventions | Replace by 10a, 10b and 10c |  |
|  | 10a |  | Who generated the random allocation sequence, who enrolled clusters, and who assigned clusters to interventions | P10-11 lines 201-208 |
|  | 10b |  | Mechanism by which individual participants were included in clusters for the purposes of the trial (such as complete enumeration, random sampling) | P10-11, p201-208 |
|  | 10c |  | From whom consent was sought (representatives of the cluster, or individual cluster members, or both), and whether consent was sought before or after randomisation | P10, lines 199-200 |
|  |  |  |  |  |
| Blinding | 11a | If done, who was blinded after assignment to interventions (for example, participants, care providers, those assessing outcomes) and how |  | P11, lines 206-208 |
|  | 11b | If relevant, description of the similarity of interventions |  |  |
| Statistical methods | 12a | Statistical methods used to compare groups for primary and secondary outcomes | How clustering was taken into account | p11, lines 210-222 |
|  | 12b | Methods for additional analyses, such as subgroup analyses and adjusted analyses |  |  |
| Results | | | |  |
| Participant flow (a diagram is strongly recommended) | 13a | For each group, the numbers of participants who were randomly assigned, received intended treatment, and were analysed for the primary outcome | *For each group, the numbers of clusters that were randomly assigned, received intended treatment, and were analysed for the primary outcome* | p11, lines 226-237 |
|  | 13b | For each group, losses and exclusions after randomisation, together with reasons | For each group, losses and exclusions for both clusters and individual cluster members | p11, lines 226-237 |
| Recruitment | 14a | Dates defining the periods of recruitment and follow-up |  | p12, line 234-237 |
|  | 14b | Why the trial ended or was stopped |  | N/A – ended at end of planned follow-up. |
| Baseline data | 15 | A table showing baseline demographic and clinical characteristics for each group | Baseline characteristics for the individual and cluster levels as applicable for each group | p12, lines 239-351 and Table 1 |
| Numbers analysed | 16 | For each group, number of participants (denominator) included in each analysis and whether the analysis was by original assigned groups | For each group, number of clusters included in each analysis | p13, lines263-269 |
| Outcomes and estimation | 17a | For each primary and secondary outcome, results for each group, and the estimated effect size and its precision (such as 95% confidence interval) | Results at the individual or cluster level as applicable and a coefficient of intracluster correlation (ICC or k) for each primary outcome | P13. ICCS line 254-261; Tables 2,3,4,5,6,7 |
|  | 17b | For binary outcomes, presentation of both absolute and relative effect sizes is recommended |  |  |
| Ancillary analyses | 18 | Results of any other analyses performed, including subgroup analyses and adjusted analyses, distinguishing pre-specified from exploratory |  | Secondary outcomes: p16 lines 237-376 |
| Harms | 19 | All important harms or unintended effects in each group (for specific guidance see CONSORT for harms^[[3]](#endnote-3)^) |  | **-** |
| Discussion | | | | P18, lines 377-443 |
| Limitations | 20 | Trial limitations, addressing sources of potential bias, imprecision, and, if relevant, multiplicity of analyses |  | p20-21, lines 445-460 |
| Generalisability | 21 | Generalisability (external validity, applicability) of the trial findings | Generalisability to clusters and/or individual participants (as relevant) | P20, lines 423-443 |
| Interpretation | 22 | Interpretation consistent with results, balancing benefits and harms, and considering other relevant evidence |  | p21-22, lines 461-472 |
| Other information | | |  |  |
| Registration | 23 | Registration number and name of trial registry |  | p5, line 80-81 |
| Protocol | 24 | Where the full trial protocol can be accessed, if available |  | p6, line 95 |
| Funding | 25 | Sources of funding and other support (such as supply of drugs), role of funders |  | p5, lines 82-83 |

** Note: page numbers optional depending on journal requirements*

**
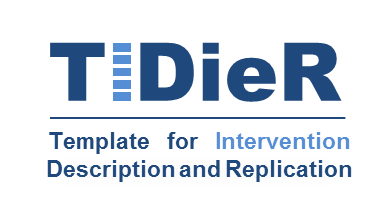
The TIDieR (Template for Intervention Description and Replication) Checklist*:**

Information to include when describing an intervention and the location of the information

| **Item number** | **Item** | **Where located **** | |
| --- | --- | --- | --- |
|  |  | Primary paper  (page or appendix  number) | Other ^†^ (details) |
|  | **BRIEF NAME** |  |  |
| **1.** | Provide the name or a phrase that describes the intervention. | p4 |  |
|  | **WHY** |  |  |
| **2.** | Describe any rationale, theory, or goal of the elements essential to the intervention. | p6/7 |  |
|  | **WHAT** |  |  |
| **3.** | Materials: Describe any physical or informational materials used in the intervention, including those provided to participants or used in intervention delivery or in training of intervention providers. Provide information on where the materials can be accessed (e.g. online appendix, URL). | p8-9 | See also protocol paper^5^  Type A from [gillian.harvey@adelaide.edu.au](mailto:gillian.harvey@adelaide.edu.au)  Type B from [BMcCormack@qmu.ac.uk](mailto:BMcCormack@qmu.ac.uk) |
| **4.** | Procedures: Describe each of the procedures, activities, and/or processes used in the intervention, including any enabling or support activities. | p8-9 |  |
|  | **WHO PROVIDED** |  |  |
| **5.** | For each category of intervention provider (e.g. psychologist, nursing assistant), describe their expertise, background and any specific training given. | p8-9 |  |
|  | **HOW** |  |  |
| **6.** | Describe the modes of delivery (e.g. face-to-face or by some other mechanism, such as internet or telephone) of the intervention and whether it was provided individually or in a group. | p8-9 | Protocol paper ^5^ |
|  | **WHERE** |  |  |
| **7.** | Describe the type(s) of location(s) where the intervention occurred, including any necessary infrastructure or relevant features. | p8 |  |

** **Authors** - use N/A if an item is not applicable for the intervention being described. **Reviewers** – use ‘?’ if information about the element is not reported/not sufficiently reported.

† If the information is not provided in the primary paper, give details of where this information is available. This may include locations such as a published protocol or other published papers (provide citation details) or a website (provide the URL).

1. Hopewell S, Clarke M, Moher D, Wager E, Middleton P, Altman DG, et al. CONSORT for reporting randomised trials in journal and conference abstracts. *Lancet* 2008, 371:281-283 [↑](#endnote-ref-1)
2. Hopewell S, Clarke M, Moher D, Wager E, Middleton P, Altman DG at al (2008) CONSORT for reporting randomized controlled trials in journal and conference abstracts: explanation and elaboration. *PLoS Med* 5(1): e20 [↑](#endnote-ref-2)
3. Ioannidis JP, Evans SJ, Gotzsche PC, O'Neill RT, Altman DG, Schulz K, Moher D. Better reporting of harms in randomized trials: an extension of the CONSORT statement. *Ann Intern Med* 2004; 141(10):781-788. [↑](#endnote-ref-3)
